# Preservation of the inferior mesenteric artery in laparoscopic nerve-sparing colorectal surgery for endometriosis

**DOI:** 10.1038/s41598-022-07237-w

**Published:** 2022-02-24

**Authors:** Marco Scioscia, Cristiano G. S. Huscher, Federica Brusca, Francesco Marchegiani, Rossella Cannone, Orsola Brasile, Pantaleo Greco, Gennaro Scutiero, Gabriele Anania, Giovanni Pontrelli

**Affiliations:** 1 Department of Obstetrics and Gynecology, Policlinico Hospital, Abano Terme, Padua Italy; 2 Department of Surgical Oncology, Robotics and New Technologies, Policlinico Hospital, Abano Terme, Padua Italy; 3 Department of Medical Science, Section of Obstetrics and Gynecology, University of Ferrara, Azienda Ospedaliero-Universitaria S.Anna, Via Aldo Moro 8, 44124 Cona, FE Italy; 4 First Surgical Clinic, Department of Surgical, Oncological and Gastroenterological Sciences, University of Padua, Padua, Italy; 5 Unit of Obstetrics and Gynecology, Department of Biomedical and Human Oncologic Science, Policlinico University of Bari, Bari, Italy; 6 Department of Medical Science, Section of General Surgery, University of Ferrara, Azienda Ospedaliero-Universitaria S.Anna, Cona, Ferrara Italy

**Keywords:** Colon, Gastroenterology, Anatomy

## Abstract

Laparoscopic rectosigmoid resection for endometriosis is usually performed with the section of the inferior mesenteric artery (IMA) distal to the left colic artery (low-tie ligation). This study was to determine outcomes in IMA-sparing surgery in endometriosis cases. A single-center retrospective study based on the analysis of clinical notes of women who underwent laparoscopic rectosigmoid segmental resection and IMA-sparing surgery for deep infiltrating endometriosis with bowel involvement between March the 1st, 2018 and February the 29th, 2020 in a referral hospital**.** During the study period, 1497 patients had major gynecological surgery in our referral center, of whom 253 (17%) for endometriosis. Of the 100 patients (39%) who had bowel endometriosis, 56 underwent laparoscopic nerve-sparing rectosigmoid segmental resection and IMA-sparing surgery was performed in 53 cases (95%). Short-term complications occurred in 4 cases (7%) without any case of anastomotic leak. Preservation of the IMA in colorectal surgery for endometriosis is feasible, safe and enables a tension-free anastomosis without an increase of postoperative complication rates.

## Introduction

Endometriosis is a benign, estrogen-dependent disease that may have a detrimental effect on the quality of life of affected women and it is characterized by ectopic localizations of endometrial-like tissue on pelvic organs and, rarely, outside the abdominal cavity^1,2^. The reported prevalence ranges between 6 to 10%^1,3^ and medical treatment (combined oral contraceptives and progestins) is considered as a first-line therapy. Surgical treatment is indicated in patients poor responder to hormonal therapy, infertility (assisted reproduction may be an option), and evidence of organ damage (mainly bowel,bladder, and ureter)^4,5^.

Bowel involvement is reported in up to 12% of patients, with rectum being the most common site^6^. In severe stages, the incidence of bowel endometriosis can be as high as 37%^7^. Bowel surgery is indicated in cases of evidence of significant stenosis^8^, symptomatic lesions (chronic pelvic pain resistant to medical therapy, dyschezia, rectal bleeding, and progressive constipation up to bowel obstruction)^9,10^, or to improve fertility^4,8,11^.

Several procedures for bowel surgery (rectal shaving, discoid excision, and colorectal resection) could be considered according to size (longitudinal and transverse diameters)^12^, appearance (plane, convex, multifocal), location (distance from the anal verge) of the nodule^13^, and previous bowel surgery^8^. The excision technique depends on the longitudinal, anterioposterior and transverse diameters of bowel endometriotic nodules. Segmental resection is needed when extimated longitudinal diameter is more than 3 cm and thickness is more than 9 mm. Discoid resection could be perfomed when extimated longitudinal diameter is less than 3 cm and thickness was 7 to 9 mm^14^. It is a complex intervention that should be performed in a multidisciplinary setting, including expert gynecologists and colorectal surgeons.

**Figure 1:**
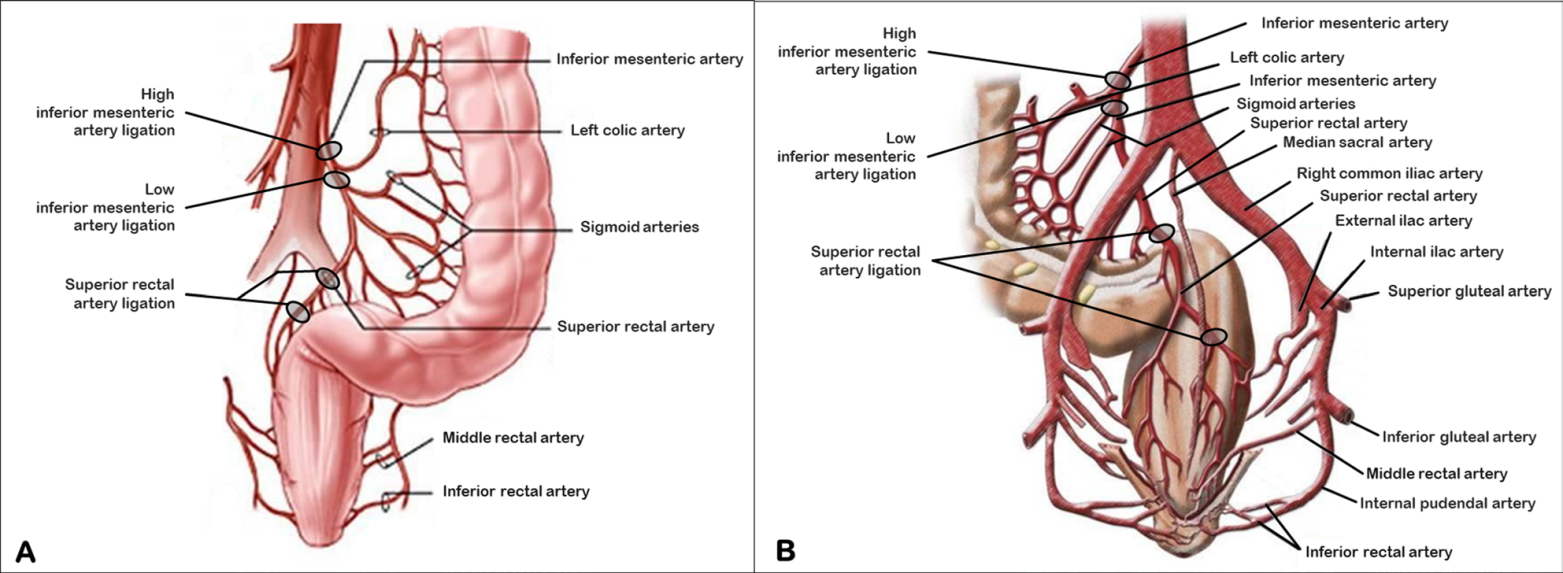
HYPERLINK "sps:id::fig1||locator::gr1||MediaObject::0" The IMA originates from the front of the abdominal aorta at the level of L3 vertebra, about 3–4 cm above the bifurcation of the abdominal aorta. Sites of artery ligations are reported in figure A and B in front and back view, respectively.

Laparoscopic rectosigmoid resection for endometriosis is usually carried out according to the procedure reported by Redwine and Sharpe^15,16^ with subsequent modifications for a nerve-sparing approach to prevent intestinal neurogenic dysfunction^8,17^. Section of the inferior mesenteric artery (IMA) distal to the left colic artery (low tie; Fig. 1) is usually performed in order to provide a tension-free anastomosis^16^. Most endometriosis nodules of the bowel involve the proximal-medial rectum or the rectosigmoid junction^7,8,18^, tracts whose vascularization is ensured by the superior rectal artery (SRA), the last branch of the IMA (Fig. 1). In the past years, IMA preservation was successfully performed for benign colorectal diseases like diverticulosis improving postoperative morbidity^19,20^. At our institution, we usually avoid clamping IMA for benign colorectal diseases and we prefer to transect the SRA whenever possible. We retrospectively reviewed our colorectal surgery cases for endometriosis, investigated perioperative outcomes and frequency of short-term complications.

## Material and methods

This was a single-center study based on the retrospective analysis of clinical notes of all women who underwent laparoscopy for endometriosis with bowel involvement between March the 1st, 2018 and February the 29th, 2020. This study was conducted in accordance with the relevant guidelines and regulations with a specific informed consent signed by all patients. The local ethics committee approved the research protocol (number 0070697, CESC code 40n/AT/20, approval on 24/11/2020) for this retrospective study on surgical data and the study was registered with UMIN Clinical Trials Registry (identification number UMIN000040625).

All patients received preoperative low-residue diet for bowel preparation and osmotic medications to clear the lumen of stool and leave gas only. Perioperative antibiotic prophylaxis (ampicillin/sulbactam plus metronidazole) was administered and discontinued after surgery, the nasogastric tube was removed soon after surgery, oral fluids within 8 h after surgery, resumption of oral semi-liquid feeding within 24 h, early mobilization, and discharge from hospital as soon as bowel function was restored.

All operations were carried out by senior consultants with high experience in performing laparoscopic nerve-sparing surgery for deep infiltrating endometriosis (gynecologists who performed surgery, namely MS and GP, came from Negrar Hospital) according to the surgical method that we previously reported^16,17,21^. Deep infiltrating endometriosis (DIE) represents the most severe form of endometriosis and it is defined as the presence of ectopic endometrial tissue infiltrating pelvic structure and organ walls including the uterosacral ligaments, rectosigmoid colon, vagina, rectovaginal septum, bladder, ureter and lateral parametrium (LP). The lateral parametrium is the retroperitoneal connective areolar tissue that extends from the uterus to the pelvic sidewall surrounding uterine vessels and enveloping lymphatic structures and nerves. The ureter divides LP into a cranial and caudal region in which the deep uterine vein represents the main anatomical landmark for the pelvic autonomic nerves^22^. When endometriosis develops as deep infiltrating nodules, extension to the parametria and posterior and lateral pelvic wall brings a considerable risk of somatic and visceral nerves infiltration or compression^17^. In this cases laparoscopic eradication of endometriosis with nerve sparing parametrial and somatic nerve decompression is needed^23^. Nerve sparing surgery has proven to be effective in preserving neurologic pelvic functions with similar disease-free intervals and clinical outcomes. The goal of nerve sparing approach is to better identify the visceral neural fibers and surgical landmarks, thereby improving the dissection of the vascular portion (pars vasculosa) from the neural portion (pars nervosa) of the parametrium and to allow the preservation of pelvic sympathetic and parasympathetic fibers of the superior hypogastric plexus, hypogastric nerves, lumbosacral sympathetic trunk, pelvic splanchnic nerves and inferior hypogastric plexus^17^. The only difference with the standard surgical procedure was that the IMA was not transected distal to the left colic artery (low tie procedure, Fig. 1) as described by Ruffo et al.^16^ (Supplementary Fig. S1 online and Supplementary Video S1 online) but the artery was preserved and transection was performed along the SRA, 1 cm above the rectal nodule (Supplementary Fig. S2 online and Supplementary Video S2 online). This technique was applied to all rectum and colorectal junction segmental resections and only in cases of sigmoid endometriosis, more proximal than rectosigmoid junction, a low tie transection of the IMA was performed.

Patient characteristics and surgical details like nodule size, number of bowel localizations, length of bowel resected, and need for ileostomy were analyzed. Short-term complications were defined as complications within 2 months of surgery. They included perioperative complications (bleeding, ureteral damage), postoperative bleeding, infection, pyrexia (≥ 38 °C), anastomotic bleeding, need for reintervention, and anastomotic stenosis. All patients were followed up for 2 months. Anatomical details (endometriosis nodule size, single or multiple nodules, and length of bowel resected) were retrieved from the histopathology reports.

Statistical significance was verified using two-way analysis of variance (ANOVA) or Kruskal Wallis test as appropriate for multiple comparisons and Fisher’s exact test for categorical variables, as well as t-tests of individual parameters (continuous variables). Continuous variables were assessed using a t-test if a normal distribution was confirmed by the method of Kolmogorov and Smirnov. Relative risk was calculated when appropriate. Data were analyzed using the GraphPad Prism software system (version 6.01 for Windows, GraphPad Software, San Diego California USA) with significance set at p < 0.05.

### Ethical standard

The study was approved by the local ethics committee (Comitato Etico per la Sperimentazione Clinica della Provincia di Padova, CESC; number 0070697, CESC code 40n/AT/20, 29/10/2020) and the study was registered with UMIN Clinical Trials Registry (identification number UMIN000040625).

## Results.

**Figure 2:**
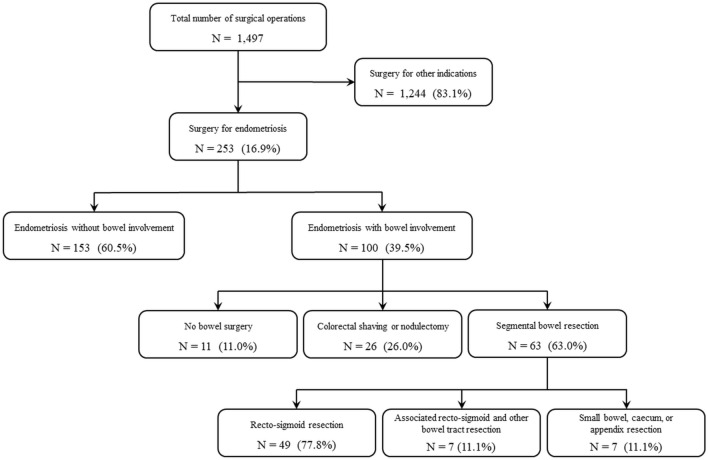
Case selection flowchart.

During the study period 1,497 major gynecological surgical procedures were performed at our unit (Fig. 2). Two hundred fifty-three (16.9%) patients underwent laparoscopy for endometriosis and their clinical characteristics are reported in Table 1. 39% of cases demonstrated bowel endometriosis (most patients, 85.8%, showed a stage III to IV according to rASRM staging system; Table 2) and 89 underwent bowel surgery. In 11 cases of bowel involvmen bowel surgery was not indicated. In Table 2 we insert all patient caracterisics with endometriosis stage III and IV, the surgical treatment performed and the incidence of complications. Colorectal shaving or nodulectomy was performed in 26 case while 63 underwent major bowel surgery. Rectosigmoid segmental resection was carried out in 56 cases (88.9%) of which 7 (12.5%) with contemporary resection of other bowel tracts (appendix, small bowel, caecum, or ileocecal valve). Seven patients (11.1%) underwent segmental resection of intestinal tracts other than rectosigmoid (Supplementary Table S1 Online). Among rectosigmoid cases, 14 (18.7%) showed multiple endometriosis nodules on the same bowel tract and in three cases the length of bowel resected was longer than 10 cm (specifically 12, 16 and 18 cm) with sigmoid involvement, therefore a low transection of the IMA was made.

**Table 1:** Patient characteristics in the two groups.

	No bowel surgery	Bowel surgery	P
	N = 164	N = 89	
Age (years, mean and SD)	35.9 ± 7.6	35.1 ± 5.9	0.36
BMI (Kg/m^2^, mean and SD)	22.5 ± 4.1	21.7 ± 3.0	0.10
Nulliparous (N, %)	128 (78.0)	82 (92.1)	< 0.01
Nulligravida (N, %)	115 (70.1)	80 (89.9)	< 0.001
Preoperative therapy (N, %)	43 (26.2)	34 (38.2)	0.06
ASA score 1–2 (N, %)	157 (95.7)	85 (95.5)	1.00
ASA score 3–4 (N, %)	7 (4.3)	4 (4.5)	1.00
Hospital stay (days, mean and SD)	3.6 ± 1.2	6.7 ± 2.4	< 0.001

**Table 2:** Surgical details in the two groups.

	No bowel surgery	Bowel surgery	P
	N = 164	N = 89	
Endometriosis stage III and IV (rASRM; N, %)	130 (73.9)	87 (97.8)	< 0.001
**Endometriosis location:**			
Anterior pelvic compartment (N, %)	38 (23.2)	43 (48.3)	< 0.001
Posterior pelvic compartment (N, %)	138 (84.1)	89 (100.0)	< 0.001
**Surgical procedures:**			
Ovary/tube surgery (N, %)	127 (77.4)	69 (77.5)	1.0
Recto-vaginal septum surgery (N, %)	88 (53.7)	75 (84.2)	< 0.001
Parametrectomy (N, %)	17 (10.4)	33 (37.1)	< 0.001
Ureterolysis (N, %)	70 (42.7)	66 (74.2)	< 0.001
Neurolysis (N, %)	18 (11.0)	41 (46.1)	< 0.001
Hysterectomy (N, %)	14 (8.5)	3 (3.4)	0.19
**Claiven-Dindo grading:**			
I (N, %)	163 (99.4)	87 (97.8)	0.28
II (N, %)	1 (0.6)	1 (1.1)	< 1.0
IIIa (N, %)	–	–	–
IIIb (N, %)	0 (0.0)	1 (1.1)	0.35
IVa (N, %)	–	–	–
IVb (N, %)	–	–	–
V (N, %)	–	–	–
Complications (N, %)	4 (2.4)	5 (5.6)	0.29

Pathology confirmed endometriosis with an infiltration of the muscular layer in all cases of shaving and segmental resections. In the group of rectosigmoid resection, nodule size was ≤ 5 cm in 92.9% (52/56) of cases and the length of bowel resected was ≤ 8 cm in 71.4% (40/46) of cases. Submucosal/mucosal layer involvement was found in 57.1% (32/56) of cases in the rectosigmoid resection group.

As for complications, no intraoperative complication was recorded, and anastomotic leak occurred in no case. The Claiven-Dindo classification system^24^ was reported in Table 2 to objective the therapy used to correct specific postoperative complications. Short-term complications were reported in 4 cases of rectosigmoid resection (4/82, 4.9%; Supplementary Table S1 Online): two cases of pyrexia without evidence of pelvic abscess treated with antibiotics, one patient with ileostomy had anastomotic stenosis and was treated with endoscopic dilatation, and one patient sought care one week after discharge for pelvic pain due to severe constipation. Multiple bowel segmental resections showed a sevenfold higher risk of complications compared to isolated rectosigmoid resections (RR 7.0, 95% CI 1.17–42.04, p = 0.03).

## Discussion

This study demonstrates that IMA-sparing surgery in laparoscopic rectosigmoid resection for endometriosis is feasible without any increase in postoperative complication rate. IMA preservation was proposed for benign colorectal diseases like diverticulosis^19,20^ and a retrospective analysis of colorectal surgery for endometriosis showed an incidence of 72% (16/22) of complications when IMA was clamped^25^. A low tie ligation of the IMA was shown to be associated with a decreased rectal blood flow in oncology cases^26^ and this is intuitive notwithstanding the anorectal vascular anastomosis system. The rectosigmoid colon receives its blood supply from the IMA branches: the sigmoid arteries to the proximal and medial sigmoid and the SRA to the rectosigmoid junction and the rectum. There exist anastomoses between the SRA with the middle rectal artery, a branch of the inferior vesical artery wich originates from the internal iliac artery. The distal part of the rectum and the anal canal receive their blood supply from anastomoses between the middle and the inferior rectal artery, which originates from the internal pudendal artery, another branch of the internal iliac artery.

**Figure 3:**
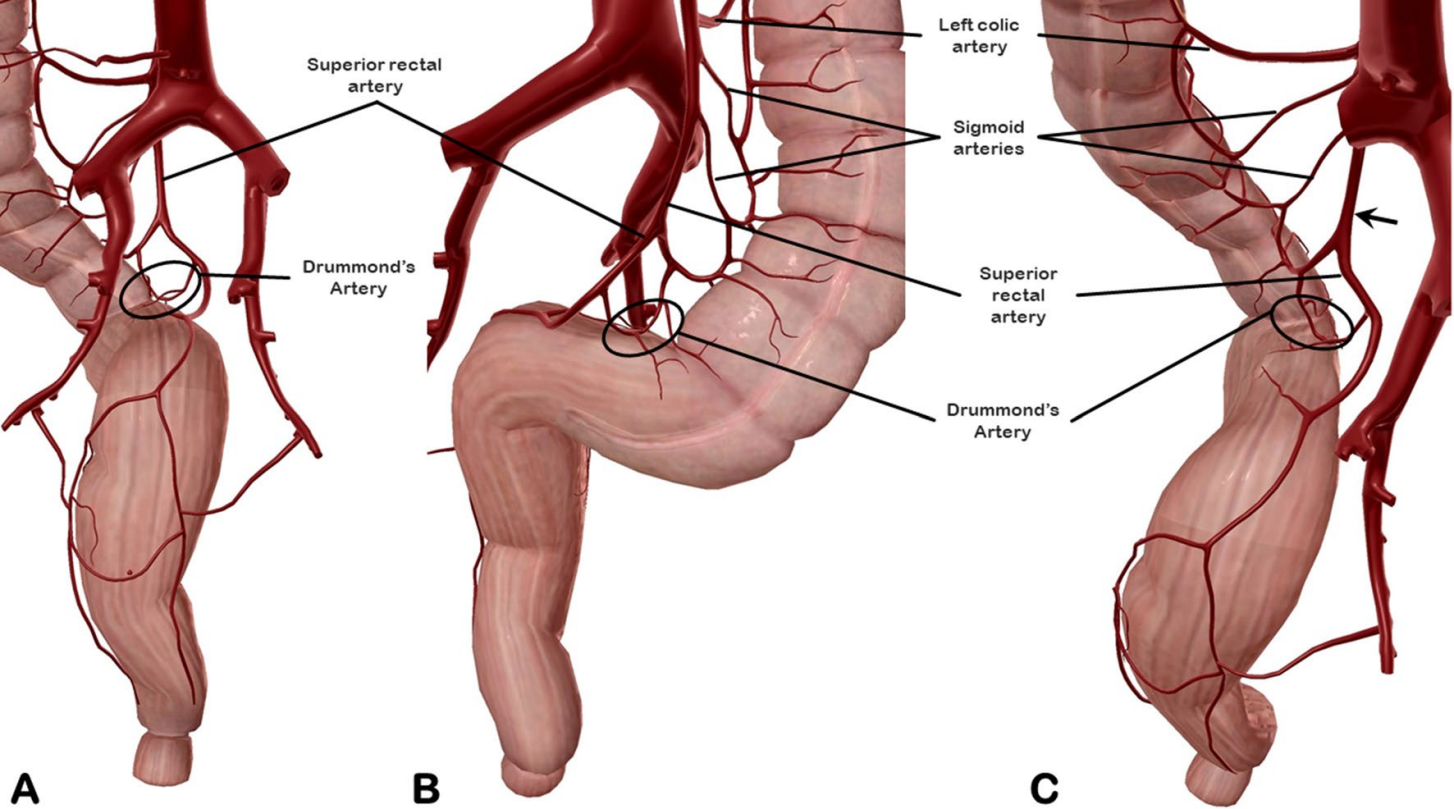
Identification of the Drummond’s artery in back (**A**), front (**B**), and back-high views (**C**).

In case of vascular transection far from the anastomotic site, as it happen in low-tie ligation of the IMA, a normal blood supply to the anastomosis comes from the middle rectal artery for the distal part of the transected rectum but a relative hypoperfusion occurs in the proximal stump before that new anastomoses develop. In fact, the anastomosis between the sigmoid artery (preserved during the low-tie procedure) and the SRA (transected by the low-tie ligation), known as the marginal artery of Drummond (Fig. 3), may sometimes be insufficient to meet the blood demands of the proximal stump for many centimeters (Sudeck’s critical point)^27^. Another observation may support this hypothesis: routinely during surgery, an hydropneumatic testing of the anastomosis is performed so, when a postoperative leak occurs, it is likely to be a failure of regenerative processes of the suture. The main causes of failed healing in any tissue is either infection or blood hypoperfusion. Altered microperfusion at the rectal anastomosis was reported to be predictive for anastomotic leakage^28^. Endovenous indocianine green (ICG) injection during rectosigmoid segmental resection is a new use of ICG to allow real-time visualization of bowel perfusion in women with endometriosis. ICG is administered through peripheral line while a near-infrared camera head enabled vision of the colorant after latency of a few seconds. It could be used to observe the ischemic area around the bowel nodule and perfusion area upstream and downstream from the lesion to select the transecting line for resection and to check bowel vascularization after direct mechanical anastomosis^29^. The anatomical variances of the Drummond’s artery (narrow or absent in 4% of cases)^27^ may justify some anastomotic leaks, a complication that occurs typically between 3 to 5 days after surgery in 2 to 4% of cases^9,10^. In cases of discoid resection for endometriosis the rectosigmoid vascular system is preserved and leakage is a rare condition^30^.

In case of SRA transection as we describe in this paper, the vascular interruption can be made 1 cm above the anastomosis verge and this may allow a better perfusion of the proximal stump than the classical low-tie ligation of the IMA. Since more than 90% of endometriosis bowel nodules involve the rectum^7,18^, the RSA transection may be more suitable than IMA ligation, leaving the latter procedure for more proximal segmental resections. Opening of pelvic peritoneum at the sacral promontorium to develop the avascular Heald’s rectrorectal plane with a backward development of the anatomical space towards the IMA origin ensures a tension-free anastomosis.

Modern management of endometriosis suggests a conservative approach that avoids surgery even in advanced cases when hormonal therapy can induce a sufficient pain relief or assisted reproduction can help to conceive^31^. Even when bowel endometriosis is found, segmental bowel resection should be limited to cases where shaving or discoid techniques are not sufficient to restore wellbeing (large nodules) or safety (severe stenosis)^32,33^. In our study cohort, most patients showed large or multiple nodules of the bowel that required segmental transection.

As most of the study on clinical and surgical outcomes in colorectal surgery for endometriosis^6,9–11,13,16,25,30^, this is a retrospective study although without selection biases as all consecutive cases during the study period were included. Another apparent bias of this study is the high number of bowel surgery in our cohort but it is due to the application of the “modern management” of endometriosis^4,8^ that avoid surgery in endometriosis patients that can be treated with hormonal therapy or may be addressed to assisted reproduction. Surgery was indicated only in severe, poor-responder patients (we had 74% of stage III–IV endometriosis cases).

One of the strengths of this single-center study is that all procedures were carried out by experienced operators using a laparoscopic nerve-sparing approach according to the Negrar method, a well-known technique for endometriosis surgery, so that results can be compared to the main series reported.

Our retrospective analysis show that SRA ligation in rectosigmoid resection for endometriosis ensures a tension-free anastomosis as IMA transection does without an increase of postoperative complication rates. We know that further studies are required to evaluate if the anastomotic leakage rate differs according to the surgical procedure but we think it is an excellent starting point for setting new research lines to know whether for endometriosis limited resections are the way to go compared to traditional bowel resection for cancer and for others benign diseases.

## Supplementary Information

Supplementary Information 1.

Supplementary Information 2.

Supplementary Information 3.

Supplementary Information 4.

Supplementary Information 5.

## Data Availability

The datasets generated during and/or analyzed during the current study are available from the corresponding author on reasonable request.

## Abbreviations

IMA: Inferior mesenteric artery
SRA: Superior rectal artery
