# Supplementary material for: Preservation of the inferior mesenteric artery in laparoscopic nerve-sparing colorectal surgery for endometriosis
Source: Sci Rep. 2022 Feb 24;12:3146. doi: 10.1038/s41598-022-07237-w (PMC8873484; doi:10.1038/s41598-022-07237-w)
Supplement: Supplementary file 1 — Supplementary Information 1. [file 41598_2022_7237_MOESM1_ESM.docx]

|  | Shaving | Recto-sigmoid resection | Recto-sigmoid and other tract bowel resection | Caecum, appendix, and/or ileum resection |
| --- | --- | --- | --- | --- |
|  | N= 26 | N= 49 | N= 7 | N= 7 |
|  |  |  |  |  |
| Nodule size (cm, Median and range) | 2.2 (0.6 – 3.8) | 3 (2 – 7) | 3 (2 – 5)* | 2.5 (2 - 6)^ |
| Multiple bowel nodules on the same tract (N, %) | 4 (15.4) | 10 (20.4) | 1 (14.3) | 2 (28.6)^§^ |
| Length of bowel resected (cm, median and range) | N/A | 5 (2.5 – 18) | 5 (4-16) | N/A |
| Ileostomy (N, %) | - | 7 (14.3) | 1 (14.3) | - |
| Anastomotic leak (N, %) | - | - | - | - |
| Complications (N, %) | - | 2 (4.1) | 2 (28.6) | 1 (14.3) |

**Supplementary Table S1.** Surgical details and outcomes according to the procedure performed. * the value refers to the recto-sigmoid nodule and in case of multiple nodules it is presented as the sum of sizes; ^ the value refers to the endometriosis nodule and in case of multiple nodules it is presented as the sum of sizes; § multiple bowel resection, e.g. appendix and ileum or caecum and ileum, while appendix and caecum were not considered as two resections.
