# Supplementary material for: Preservation of the inferior mesenteric artery in laparoscopic nerve-sparing colorectal surgery for endometriosis
Source: Sci Rep. 2022 Feb 24;12:3146. doi: 10.1038/s41598-022-07237-w (PMC8873484; doi:10.1038/s41598-022-07237-w)
Supplement: Supplementary file 2 — Supplementary Information 2. [file 41598_2022_7237_MOESM2_ESM.docx]

Brief title video S1: Laparoscopic transection of the inferior mesenteric artery

Legend video S1: Laparoscopic low vascular transection of the inferior mesenteric artery in colorectal surgery for endometriosis
