# Supplementary figures and images for: Preservation of the inferior mesenteric artery in laparoscopic nerve-sparing colorectal surgery for endometriosis
Source: Sci Rep. 2022 Feb 24;12:3146. doi: 10.1038/s41598-022-07237-w (PMC8873484; doi:10.1038/s41598-022-07237-w)

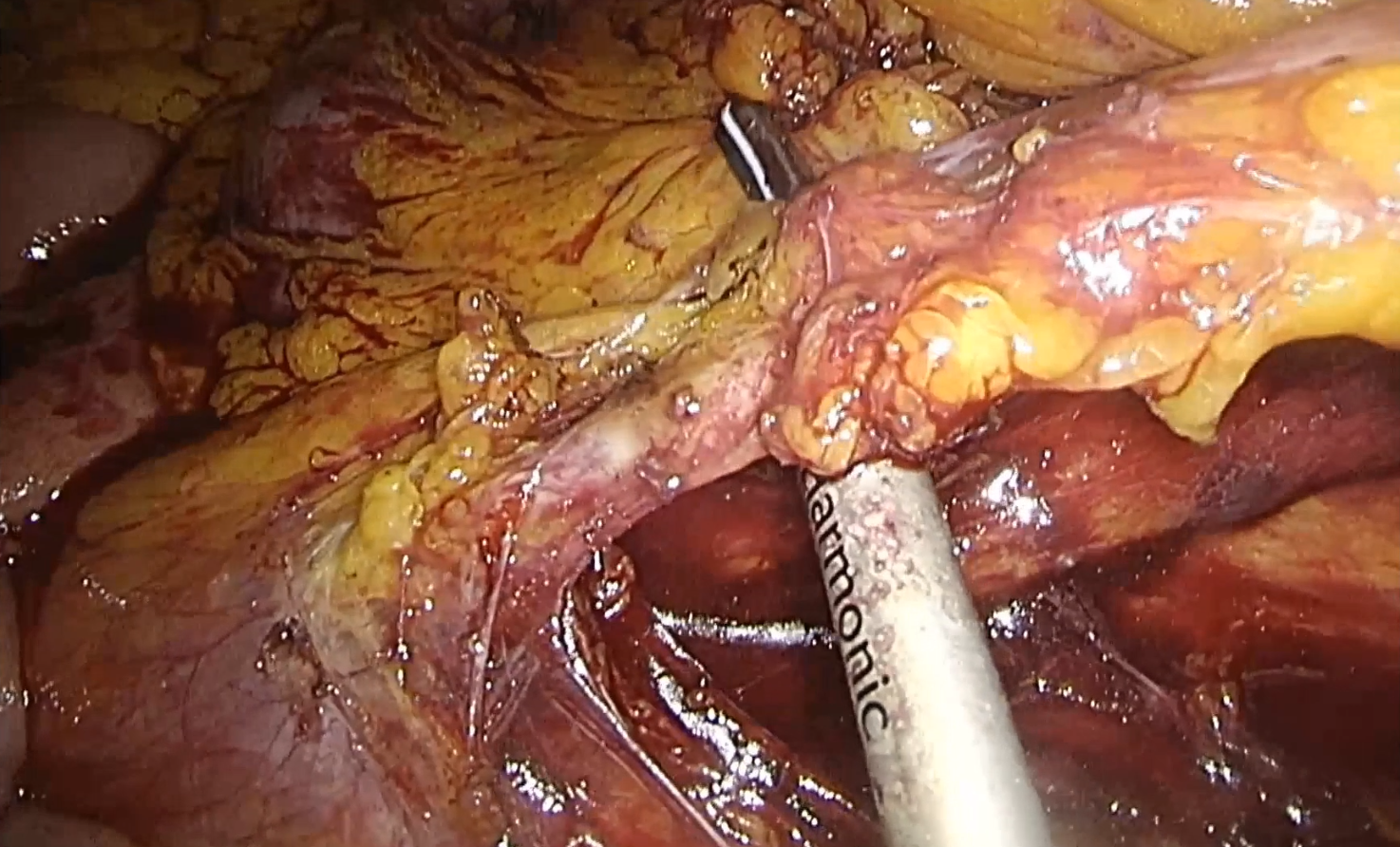

Supplement: Supplementary file 3 — Supplementary Information 3. [file 41598_2022_7237_MOESM3_ESM.tif]

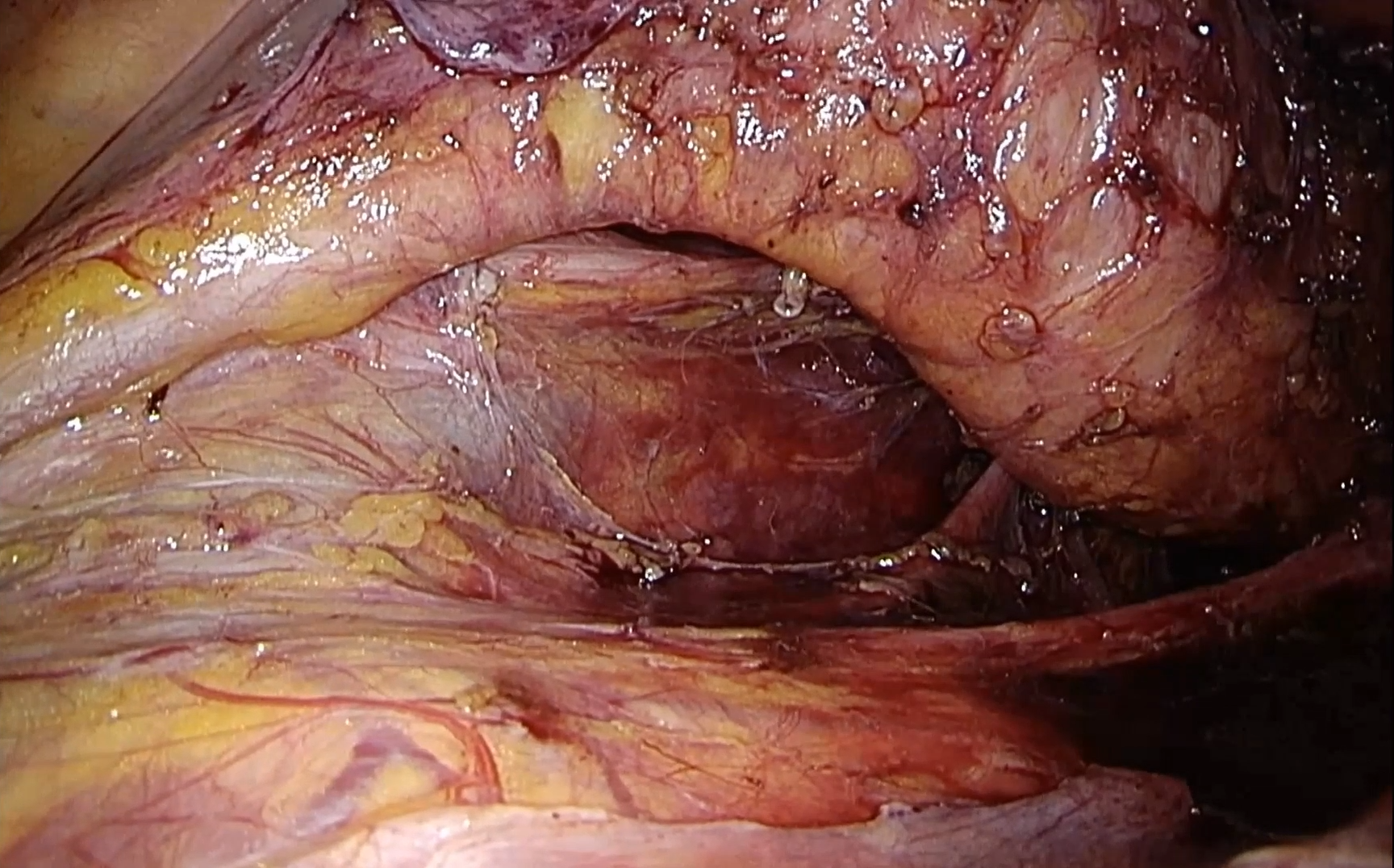

Supplement: Supplementary file 5 — Supplementary Information 5. [file 41598_2022_7237_MOESM5_ESM.tif]
