# Supplementary material for: Preservation of the inferior mesenteric artery in laparoscopic nerve-sparing colorectal surgery for endometriosis
Source: Sci Rep. 2022 Feb 24;12:3146. doi: 10.1038/s41598-022-07237-w (PMC8873484; doi:10.1038/s41598-022-07237-w)
Supplement: Supplementary file 4 — Supplementary Information 4. [file 41598_2022_7237_MOESM4_ESM.docx]

Brief title video S2: Laparoscopic vascular transection of the superior rectal artery

Legend video S2: Preservation of the inferior mesenteric artery and laparoscopic vascular transection of the superior rectal artery in colorectal surgery for endometriosis
